# A case report of unusually long lag time between immunotactoid glomerulopathy (itg) diagnosis and diffuse large B-cell lymphoma (DLBCL) development

**DOI:** 10.1186/s12882-016-0349-9

**Published:** 2016-09-29

**Authors:** Aditi Khandelwal, Martina A. Trinkaus, Hassan Ghaffar, Serge Jothy, Marc B. Goldstein

**Affiliations:** 1 Department of Medicine, University of Toronto, Suite RFE 3-805, 200 Elizabeth Street, Toronto, ON M5G 2C4 Canada; 2 Department of Laboratory Medicine, St. Michael’s Hospital and Department of Laboratory Medicine and Pathobiology, Univesity of Toronto, 30 Bond Street, Toronto, ON M5B 1W8 Canada

**Keywords:** Immunotactoid glomerulopathy, Lymphoproliferative disorder, Monoclonal gammopathy of renal significance

## Abstract

**Background:**

Immunotactoid glomerulopathy (ITG) is a rare cause of proteinuria characterized by organized microtubular deposits in the glomerulus. ITG has been associated with underlying lymphoproliferative disorders and any renal impairment may be reversible with treatment of the concomitant hematologic malignancy. This case is the first reported in literature where diffuse large B cell lymphoma developed two years following the initial ITG diagnosis.

**Case presentation:**

A 55-year-old woman with a history of well-controlled diabetes mellitus and thalassemia trait presented with proteinuria (830 mg/day) in 2010. Initially, she was managed with renin-angiotensin-aldosterone-system blockade. In 2012, the proteinuria worsened (4.3 g/day) and a renal biopsy showed immunotactoid glomerulopathy (Fig. 1). Despite extensive work up, no lymphoproliferative disorder was initially found. In January 2014, the patient presented with a soft-palate mass found on biopsy to be diffuse large B-cell lymphoma. She received 6 cycles of R-CHOP, 4 cycles of high dose methotrexate chemotherapy for CNS prophylaxis and 30 Gy of Intensity Modulated Radiation Therapy. Follow-up revealed complete remission of diffuse large B-cell lymphoma and resolution of proteinuria from the ITG.

**Conclusion:**

As we recognize that patients with ITG may develop hematopoietic neoplasms, close long-term monitoring is important. Moreover, treatment of the lymphoproliferative disorder can allow for complete remission of ITG.

## Background

Immunotactoid Glomerulopathy (ITG) is a rare cause of proteinuria characterized by Congo-red negative microtubular deposits in the glomerulus, which are often monoclonal [1, 2]. There has been controversy in recent years regarding the distinction between fibrillary glomerulonephritis (FGN) and ITG, due to lack of clinical significance and overlap in the size of deposited fibrils [3]. However, many recent studies have shown an important correlation between monoclonal gammopathy or lymphoproliferative disorders (LPD) and organized tubular deposits in the glomerulus as seen in ITG [4–7]. In fact, in a study of 16 ITG patients by Nasr and colleagues (2012) [6], there was a serum-M spike in 63 % and a hematologic malignancy in 38 % of the patients. As seen in our case, multiple studies have found remission of the nephrotic syndrome with therapy directed against the underlying LPD [2, 6]. Thus, it is important to distinguish ITG from FGN and direct investigations towards identifying an underlying LPD, allowing for effective treatment [8].

Monoclonal gammopathy accompanying renal impairment is increasingly being recognized as an independent entity, and called monoclonal gammopathy of renal significance (MGRS) [9]. In patients with MGRS due to ITG, the current recommendation is to perform thorough investigations to identify an underlying LPD at the time of diagnosis [8]. In a survey of English language literature reporting incidence of LPD in ITG, the longest duration between initial ITG diagnosis and hematopoietic malignancy is 8 months [10] (Table 1). Most cases have either existing LPD or are diagnosed concurrently with ITG (Table 1). We report a case of ITG where the patient developed a diffuse large B-cell lymphoma (DLBCL) over twenty months after the initial diagnosis. There is little guidance regarding the required duration for LPD surveillance in ITG patients. In fact, there is a developing opinion that one might institute therapy for MGRS at the time of the initial diagnosis, but the initial therapy, in the absence of a specific neoplastic cellular diagnosis is based on the probability of a given neoplastic process developing [8].

**Table 1:** Incidence and timing of hematologic malignancy onset in patients diagnosed with Immunotactoid glomerulonephropathy

Study/Case Report	Number of ITG patients included	Incidence of monoclonal spike	Incidence of hematologic malignancy	Onset of hematologic malignancy
Pronovost et al. 1996 [11]	22	-	9/22	Not described
Rosenstock JL et al., 2003 [7]	6	4/6	2/6	Before or concomitant to ITG diagnosis
Bridoux et al., 2002 [2]	14	5/14	7/14	Before or concomitant to ITG diagnosis
Nasr SH et al., 2012 [6]	16	10/16 (63 %)	6/16 (38 %)	Ranged from 6 years prior to concomitant diagnosis
Fogo, A. et al., 1993 [12]	6	3/6	1/6	unclear
Jacobson E et al., 2004 [10]	1	1	1	8 months post-ITG diagnosis
Jabur WL et al., 2008 [13]	1	1	1	concurrently
Matsushita et al., 2005 [14]	1	1	1	5 months prior to ITG
Castro JE et al., 2012 [15]	1	1	1	5 years prior to ITG
Vigil et al., 1998 [16]	1	1	1	5 months post-ITG diagnosis
Witzens-Harig M et al., 2007 [17]	1	1	1	3 years prior to ITG

## Case presentation

A 55-year-old woman with a history of well-controlled diabetes mellitus and alpha-thalassemia trait presented with proteinuria in 2010. At the time, her medications included metformin, sitagliptin, acarbose, and atorvastatin.

On initial exam, her blood pressure was 130/70 mmHg with a pulse of 78 beats per minute. Apart from a 3/6 systolic ejection murmur, the remainder of the physical exam was unremarkable There was no abdominal organomegaly, no lymphadenopathy and no peripheral edema. Laboratory studies included normal serum electrolytes, a blood urea of 3.7 mmol/L and creatinine of 58 umol/L. Serum biochemistry showed normal electrolytes. The HDL was 1.35 mmol/L and the LDL was 2.46 mmol/L. TSH was normal at 0.92 mmol/L and complement levels were normal. ANA was elevated at 7.3 (normal <1.0). A urinalysis was positive for blood, but negative for protein. There were 2–4 red cells and white cells per high power field and the sediment contained hyaline casts several containing both red and white blood cells. A 24 h urine collection contained 830 mg of protein.

Despite a normal blood pressure and renal chemistries, her urinalysis suggested an underlying proliferative form of glomerulonephritis with the presence of red cells and hyaline casts containing cells. Hypothesized differential diagnoses included diabetic nephropathy or a mild proliferative glomerulonephritis, such as IgA nephropathy. Trandolapril, a renin-angiotensin-aldosterone system blocker, was initiated to minimize proteinuria with the plan to titrate dosage to blood pressure and a protein excretion rate below 500 mg per day.

The patient was lost to follow-up until February 2012 when she was re-referred for worsening kidney function and proteinuria. When reviewed again by the nephrologist, physical examination revealed a blood pressure of 129/80 mmHg lying with a pulse of 78 beats per minute and 116/79 mmHg sitting with a pulse of 80 beats per minute. Chest was clear. Heart sounds S1 and S2 were present with a grade 3/6 systolic ejection murmur, unchanged from previous examination. There was no organomegaly, no lower extremity edema, and no skin lesions.

Renal indices revealed an elevated blood urea at 8.7 mmol/L, creatinine of 84 umol/L, serum albumin reduced to 28 g/L and mild elevations of her uric acid (3.78 mmol/L), calcium (2.63 mmol/L) and phosphorus levels (1.6 mmol/L). Her hemoglobin A1C was 6.2 %. A random urine sample contained 2.5 g/L of protein and 6.5 mmol/L of creatinine. The sediment contained 2-4 white cells, and red cells and many granular and hyaline casts, many containing red and white blood cells. The 24 h urine collection showed an increase in proteinuria to 4.3 g per day. The urine culture grew no organisms. Hepatitis B surface antigen was negative, Hepatitis B surface antibody was positive and core antibody was positive in keeping with a previous infection with acquired immunity. Hepatitis C antibody was negative. The parathyroid and thyroid hormone levels remained normal. Protein electrophoresis showed a discrete band in the gamma region, with immunofixation confirming an IgG kappa monoclonal protein. The complement components were normal and cryoglobulins were absent. ANA was elevated at 8.6 (abnormal >1.0). The autoimmune workup was negative for anti-Smith, Anti-SM-RNP, Anti Scl-70, Anti-Jo, Anti-DNA, CCP Antibody, and Rheumatoid factor. Her CBC revealed a worsening microcytic anemia with hemoglobin of 91 g/L and a lower MCV of 61.5 fL. The ESR was elevated at 66. On endoscopy, she was found to have Barrett’s oesosphagus and a proton pump inhibitor was started.

**Fig. 1:**
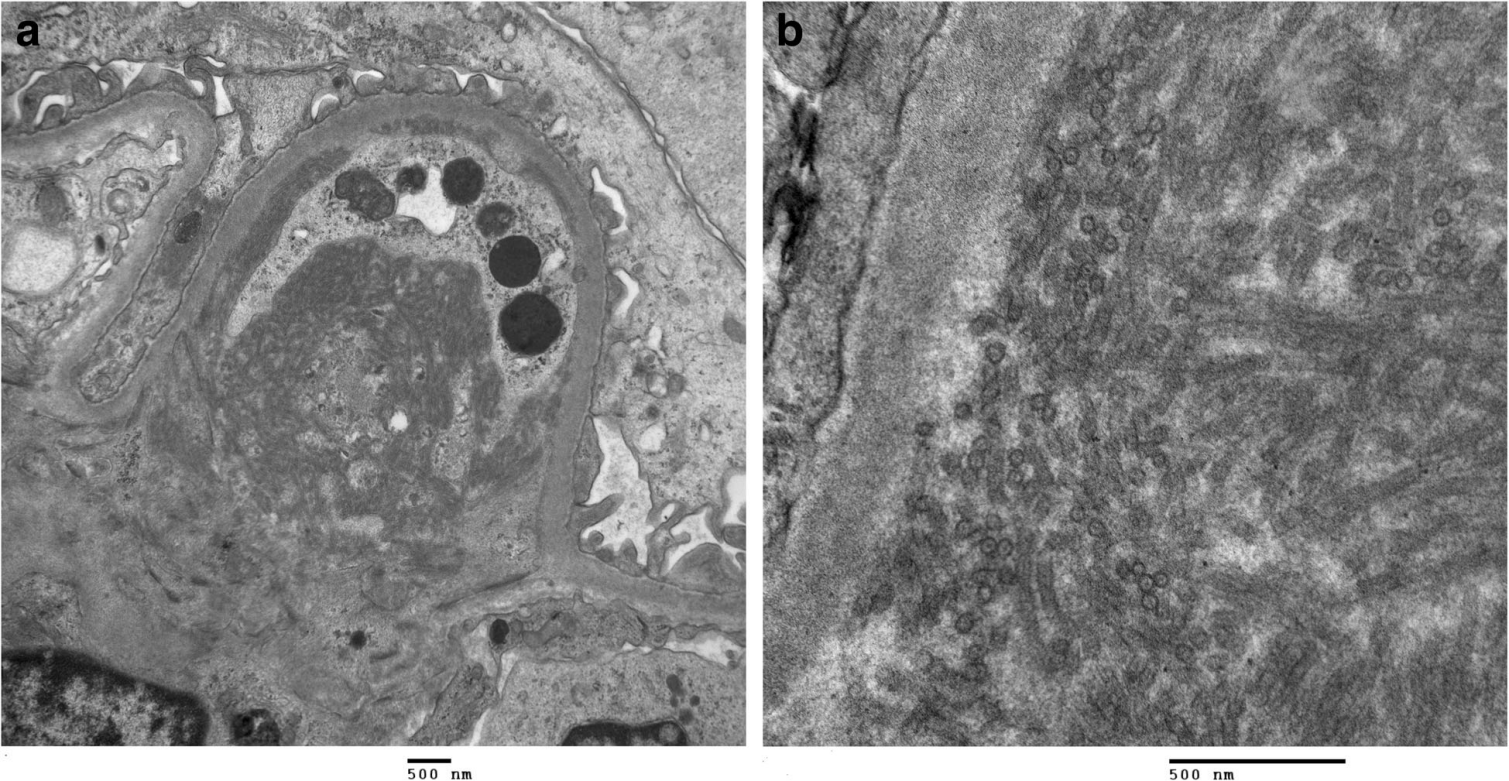
Kidney biopsy. **a** Kidney biopsy histology with H&E staining shows an increased lobular pattern with mesangial expansion in the glomeruli. **b** Electron microscopy images at 15000x and 60000x magnification reveals broad tubular structures located in subendothelial and mesangial areas of the glomeruli, measuring 30 nm in diameter. The kidney biopsy was consistent with ITG

Due to worsening proteinuria, a renal biopsy (Fig. 1) was performed. Of the 28 glomeruli examined, 2 glomeruli were globally sclerosed. Most glomeruli had an increased lobular pattern associated with mild endocapillary hypercellularity. Small aggregates of polymorphonuclear leukocytes were present in some glomeruli. No crescents were seen. The mesangial matrix and the capillary wall thickness were increased (Fig. 1a). Congo red stain was negative for amyloid. There was mild focal interstitial fibrosis. Arteries showed focal intimal and medial thickening. Tubules were atrophic in small focal areas. Immunofluorescence showed trace mesangial smudgy staining for IgM, C3 and fibrinogen. Electron microscopy (Fig. 1b) showed mostly parallel tubular structures located in subendothelial and mesangial areas of the glomeruli. The glomerular basement membrane was mildly thickened in focal areas. The overall pathologic diagnosis was ITG.

Given the diagnosis of ITG, a hematologic workup was conducted to investigate a possible underlying LPD. Repeat serum electrophoresis revealed two discrete bands in the gamma region with two M proteins quantified at 2.8 g/L and 0.7 g/L. Quantitative immunoglobulins were 12.5 g/L, 1.84 g/L and 2.77 g/L for IgG, IgA and IgM respectively. Free kappa was at 64.8 mg/L and free lambda was 32.9 mg/L with an abnormal ratio 1.97. Urine immunofixation showed free kappa light chains. Computed tomography imaging in August 2012 revealed two elongated nodes in the external iliac areas measuring 0.6 cm by 2.6 cm without any other lymphadenopathy or concerning lesions. At reassessment, she had a 4 % positive cryofibrinogen level. Despite two lymph node biopsies and two bone marrow biopsies there was no evidence of LPD on flow cytometry and histology.

In January 2014, 2 years after the initial diagnosis of ITG, the patient presented with a 2-week history of a painless, enlarging left palatal mass. This mass was pink, smooth and firm with no redness, warmth or ulceration. On biopsy, the lesion was found to be a Diffuse Large B cell Lymphoma (DLBCL). The morphology was not consistent with other aggressive lymphoma subtypes such as plasmablastic or Burkitt lymphoma. By immunohistochemistry, lymphocytes were positive for CD20, BCL-2, BCL-6 (weak), and MUM-1 and negative for CD 3, CD 10, CD 5 and CD 30. Ki-67 stains revealed a proliferation rate of 90–95 %. Fluorescence in-situ hybridization (FISH) revealed no rearrangements in C-MYC, BCL-2 and BCL-6.

This patient was diagnosed with a stage IV diffuse large B cell lymphoma (DLBCL) with imaging confirming nasopharyngeal and palatal involvement. She received 6 cycles of R-CHOP combined with 4 cycles of high dose methotrexate chemotherapy for central nervous system prophylaxis. She subsequently received total dose of 30 Gy to the nasopharynx area with Intensity Modulated Radiation Therapy (IMRT).

Seven months post-R-CHOP and IMRT, she was in complete remission of her DLBCL confirmed with CT and Positron Emission Tomography (PET) imaging. Moreover, her urinalysis was negative for blood and protein with rare cells and one cellular cast on microscopy. The 24-h urine collection had 102 mg of protein (Table 2). This indicated a complete remission of the proteinuria from ITG.

**Table 2:** Indices of renal function at different stages including pre-diagnosis (2010), diagnosis (2012), post-treatment (2015)

Test	2010	2012	2015
Urea (mmol/L)	3.7	8.7	8.4
Creatinine (umol/L)	58	84	77
Estimated glomerular filtration rate^a^ (mL/min/1.73 m^2)	99.5	64.4	70.5
Urine protein excretion (mg/24 h)	830	4300	102
Urine creatinine excretion (mmol/24 h)	5.9	6.5	6.8

Note: Conversion factor for units: urea in mmol/L to mg/dL, *x*2.80;creatinine in umol/L to mg/dl, x0.0113

^a^Modification of Diet in Renal Disease (MDRD) equation based GFR calculation

## Conclusions

Patients with MGRS due to ITG may inevitably develop malignant hematopoietic neoplasms. Pharmacologic treatment can be effective when directed towards a definitive underlying pathologic diagnosis [2, 6, 7]. As CLL is the most commonly recognized underlying LPD in ITG, some experts have suggested treating with agents known to be effective in CLL [8]. However, there is a heterogeneity in underlying LPD diagnoses. Our patient, for instance, had DLBCL and CLL treatment would have been non-curative. Moreover, Nasr et al. [6] found that amongst 16 patients with ITG, 3 had CLL but 2 had lymphoplasmacytic lymphoma and another 2 had myeloma. Bridoux et al. [2] prospectively studied 14 ITG patients with 6 patients diagnosed with CLL and 1 with small lymphocytic B-cell lymphoma. Most LPD diagnoses were either made prior to or concurrent with MGRS recognition. ITG remission was achieved in most patients treated with chemotherapy directed against the LPD [2, 6]. Heterogeneity in treatment and toxicity of chemotherapy agents prevents a clear ability to predict which agents may be most effective without a definitive pathologic diagnosis. As such, close monitoring for disease progression with excisional biopsies of lymph nodes or full cytogenetic and molecular testing of bone marrow specimens is recommended.

This case highlights the importance of continued vigilance in patients diagnosed with ITG for development of a LPD. Our patient initially presented with MGRS due to ITG. There was likely an insidious clonal process which declared itself as a DLBCL after a prolonged lag time. However, the possibility of MGUS converting into DLBCL or co-occurrence of a novel DLBCL cannot be difinitively excluded. Even though most ITG patients who do develop LPDs are diagnosed before or concurrently, there are a few who may develop LPD late. Due to minimal long-term follow-up data for ITG patients, it is difficult to determine a specific follow-up duration. However, close follow-up is essential as treatment of the underlying LPD can allow complete remission of ITG. Perhaps, with time, evidence may develop supporting a specific therapeutic regimen which may be employed at the time of the diagnosis of the MGRS even in the absence of a specific LPD diagnosis.

## Abbreviations

DLBCL: Diffuse large B-cell lymphoma
FGN: Fibrillary glomerulonephritis
ITG: Immunotactoid glomerulopathy
LPD: Lymphoproliferative disorders
